# A Shared Epitope of Collagen Type XI and Type II Is Recognized by Pathogenic Antibodies in Mice and Humans with Arthritis

**DOI:** 10.3389/fimmu.2018.00451

**Published:** 2018-04-12

**Authors:** Dongmei Tong, Erik Lönnblom, Anthony C. Y. Yau, Kutty Selva Nandakumar, Bibo Liang, Changrong Ge, Johan Viljanen, Lei Li, Mirela Bãlan, Lars Klareskog, Andrei S. Chagin, Inger Gjertsson, Jan Kihlberg, Ming Zhao, Rikard Holmdahl

**Affiliations:** ^1^Department of Medical Biochemistry and Biophysics, Section for Medical Inflammation Research, Karolinska Institute, Stockholm, Sweden; ^2^Department of Pathophysiology, Key Laboratory for Shock and Microcirculation Research of Guangdong, Southern Medical University, Guangzhou, China; ^3^Medical Immunopharmacology Research, School of Pharmaceutical Sciences, Southern Medical University, Guangzhou, China; ^4^Department of Chemistry—Biomedical Center, Section of Organic Chemistry, Uppsala University, Uppsala, Sweden; ^5^Department of Physiology and Pharmacology, Karolinska Institute, Stockholm, Sweden; ^6^Department of Medical Biochemistry and Biophysics, Section of Vascular Biology, Karolinska Institute, Stockholm, Sweden; ^7^Rheumatology Unit, Department of Medicine, Karolinska Institute, Karolinska University Hospital, Stockholm, Sweden; ^8^Institute for Regenerative Medicine, Sechenov First Moscow State Medical University, Moscow, Russia; ^9^Department of Rheumatology and Inflammation Research, University of Gothenburg, Gothenburg, Sweden

**Keywords:** rheumatoid arthritis, arthritis, autoantibody, collagen XI, CII, cross-reactive

## Abstract

**Background:**

Collagen XI (CXI) is a heterotrimeric molecule with triple helical structure in which the α3(XI) chain is identical to the α1(II) chain of collagen II (CII), but with extensive posttranslational modifications. CXI molecules are intermingled in the cartilage collagen fibers, which are mainly composed of CII. One of the alpha chains in CXI is shared with CII and contains the immunodominant T cell epitope, but it is unclear whether there are shared B cell epitopes as the antibodies tend to recognize the triple helical structures.

**Methods:**

Mice expressing the susceptible immune response gene *Aq* were immunized with CII or CXI. Serum antibody responses were measured, monoclonal antibodies were isolated and analyzed for specificity to CII, CXI, and triple helical collagen peptides using bead-based multiplex immunoassays, enzyme-linked immunosorbent assays, and Western blots. Arthritogenicity of the antibodies was investigated by passive transfer experiments.

**Results:**

Immunization with CII or CXI leads to a strong T and B cell response, including a cross-reactive response to both collagen types. Immunization with CII leads to severe arthritis in mice, with a response toward CXI at the chronic stage, whereas CXI immunization induces very mild arthritis only. A series of monoclonal antibodies to CXI were isolated and of these, the L10D9 antibody bound to both CXI and CII equally strong, with a specific binding for the D3 epitope region of α3(XI) or α1(II) chain. The L10D9 antibody binds cartilage *in vivo* and induced severe arthritis. In contrast, the L5F3 antibody only showed weak binding and L7D8 antibody has no binding to cartilage and did not induce arthritis. The arthritogenic L10D9 antibody bound to an epitope shared with CII, the triple helical D3 epitope. Antibody levels to the shared D3 epitope were elevated in the sera from mice with arthritis as well as in rheumatoid arthritis.

**Conclusion:**

CXI is immunologically not exposed in healthy cartilage but contains T and B cell epitopes cross-reactive with CII, which could be activated in both mouse and human arthritis and could evoke an arthritogenic response.

## Introduction

Rheumatoid arthritis (RA) is a chronic inflammatory disease with a prevalence of around 0.5–1% in general population (1). B cells are likely to be important for arthritis progression as CD20+ B cell depletion treatment with rituximab improves the disease symptoms, especially in seropositive RA patients (2). Several mouse models that mimic RA are dependent on B cells and autoantibodies, for example, disease development in type II collagen-induced arthritis (C^II^IA), was abrogated in B cell-deficient mice (3). Administration of monoclonal antibodies against either collagen II (CII), or other joint proteins, such as cartilage oligomeric matrix protein (4), can induce arthritis in naïve mice (5).

Collagen XI (CXI) is a minor component of articular cartilage, with the percentage ranging from 3 to 10% depending on the maturation stages (6), whereas CII constitutes 80–85% of the total cartilage. Both CXI and CII are triple helical molecules. CXI is a heterotrimer with three distinct α chains [α1(XI), α2(XI), α3(XI)], while CII is a homotrimer with three identical α chains [α1(II)]. Interestingly, α3(XI) is encoded by the same gene as α1(II), but with a higher degree of glycosylation modifications (7).

The collagen fibrillar network allows cartilage to entrap proteoglycans and provides tensile strength to the tissue; therefore, it is essential for normal cartilage development. Cartilage-specific fibrils contain a mixture of CII, CXI and CIX molecules. There are two different populations of fibrils in normal cartilage that could be distinguished according to their width (thin: 20 nm diameter, thick: 40 nm diameter). It has been shown that CXI molecules exist only in thin cartilage fibrils, which are constructed by a core of four micro-fibrils of which two are CII and the remaining two are CXI surrounded by a ring of 10 CII micro-fibrils (8). Cho/Cho mouse (autosomal recessive chondrodysplasia) having a mutation in the gene encoding for α1(XI) chain, and human Stickler/Marshall syndrome in which mutations in the genes encoding for α1(II), α1(XI), or α2(XI) were observed, have defect in the initiation of thin fibrils formation, and, therefore, develop abnormal cartilage and display osteochondrodysplasia phenotype (9–11).

Collagen XI molecules are suggested to be hidden within the cartilage fibrils, and thus most likely are not accessible to antibodies (8, 12). Nevertheless, antibody responses to both CXI and CII have been detected in sera from patients with established RA (13, 14). In the pristane-induced arthritis model (PIA), rats with a particular MHC allele exhibit antibody responses to CXI in both the acute and chronic phases of the disease; while the antibody responses to CII were detected only during the acute phase of the disease (13, 15). The anti-CXI antibody response could possibly be due to the sequence similarity between the two proteins and/or due to exposure of CXI because of the destruction of collagen fibrils in the peripheral joints during the later phase of arthritis. In fact, it has been shown that immunization of DA rats with homologous CXI leads to chronic and relapsing arthritis (15). However, in mice, conflicting results were observed on the pathogenicity of CXI though elevated antibody titers against CXI were observed (16, 17). The difference in the purity of the collagen preparations used in these studies might explain the observed variation in the results.

The importance of CXI in normal cartilage fibril formation, together with the structure and sequence similarity between CXI and CII led us to investigate the functional relevance of shared epitopes of CXI/CII both in mice and humans.

## Materials and Methods

### Animals

DBA/1J, BQ.Cia9i, B10.RIII male, age-matched mice were used in current study. BQ.Cia9i congenic mice were generated by introgressing *NOD* gene fragment (170.9–173.4 Mbp) containing the cluster of *FcγR* genes on the B10.Q genetic background and are found to be highly susceptible to antibody initiated inflammation (18, 19). Similarly, B10.RIII is an MHC congenic strain with the *H2^r^* haplotype prone to antibody-induced arthritis (20). DA/OlaHsd rats were originated from Harlan Europe (The Netherlands) (21). All the animals were kept and bred in the Medical Inflammation Research animal facility, Karolinska Institute, which is specific pathogen free (Felasa II), and climate-controlled environment with a 14 h light/10 h dark cycles. All the animals were housed in intra-cage ventilated polystyrene cages containing wood shavings, fed standard rodent chow and *water ad libitum*. This study was carried out in accordance with the recommendations of N490/12, N35/16 (Stockholm ethical committee) and 86/609/EEC guidelines (European Community Council Directive). The protocols were approved by local animal welfare authorities.

### Collagen Purification

Rat CII (rCII) and rat CXI (rCXI) were prepared from the Swarm rat chondrosarcoma grown in male DA rats by neutral salt extraction followed by salt precipitation and DEAE-cellulose chromatography (22, 23). Briefly, tumor tissue was homogenized and extracted with 1.0 M NaCl/50 mM Tris/pH 7.5 buffer. The polysaccharides and negatively charged proteins were removed and differential salt extraction procedure was used to separate different collagens, 0.9 M NaCl for CII and 1.2 M for CXI. The bovine CXI (bCXI) was extracted from joint cartilage and further purified as described above. All the collagens were lyophilized, weighed, dissolved, and stored in 0.1 M acetic acid until used. The purity of the respective fractions was analyzed using SDS-PAGE.

### Peptide Design and Synthesis

In order to identify the cross-reactive epitopes between α3(XI) and α1(II) in CXI antibodies, a library of human CII triple helical peptides as described previously was used (24). One subset of the CII peptides library was synthesized by GL Biochem, the 24 amino acids of interest (8 triplets) are flanked by 5 N-terminal and 5 C-terminal GPO repeats that help maintain its triple helical conformation. Of the C-terminal 9 amino acids of interest, all but the last peptide overlapping with the N-terminal 9 amino acids of the next peptide. Thus, each successive peptide sequence advances 15 amino acids along the triple helical sequence of human CII. All other triple helical peptides used in the present study were synthesized as covalently linked homotrimeric peptides with 5 N-terminal and 5 C-terminal GPO repeats. In these peptides, two of the α chains are carboxy-terminally linked to the amino groups of two consecutive lysine residues added to the sequence of the third chain (25). The cyclic peptides used as controls each have 15 amino acids derived from human CII. Two cysteine residues were added at both the terminal to achieve a cyclic structure. At the N-terminus of the peptide, we included biotin using the flexible linker aminohexanoic acid (Ahx). All the designed cyclic peptides were synthesized by WuXi Apptech (Wuxi, China).

### T Cell Recall Assay

96-well culture plates were coated with antigens (bCXI, rCII, denatured bCXI, denatured rCII, GalCII259-273, or nCII259-273) in 100 µl culture medium (5% FCS, 10 mM HEPES, penicillin/streptomycin) at a concentration range of 0–20 µg/ml. Mouse splenocytes at a concentration of 5 × 10^5^ cells/well in 50 µl volume were added to the culture plates coated with the antigen. T cell hybridomas HCQ3 and HCQ4, as described in detail previously (26), 5 × 10^4^ cells/well in 50 µl volume, were added to the corresponding culture plates. IL-2 concentration in the supernatant was determined after 24 h (in the case of peptides) or 48 h (in the case of proteins).

### Generation of CXI-Specific Monoclonal Antibodies

Anti-CXI monoclonal antibody-producing hybridomas were generated and characterized following the protocol described earlier (27). Briefly, 2 months old DBA/1J male mice were immunized intradermally at the base of the tail with 100 µg of bCXI dissolved in 0.1 M acetic acid and emulsified in an equal volume of complete Freund’s adjuvant (CFA) (Difco, MI, USA) on day 0, followed by a booster with 50 µg of bCXI emulsified in incomplete Freund’s adjuvant (IFA; Difco) on day 21. Three days after the booster dose, inguinal lymph cells were harvested and fused with NSO-bcl2 myeloma cells and cultured for 14 days in complete DMEM/HAT medium. Cells secreting anti-bCXI antibodies were selected using bCXI coated enzyme-linked immunosorbent assay (ELISA) plates, subcloned for five times by limiting dilution method and expanded for antibody production. Monoclonal antibodies were purified from cell culture supernatant using Gamma-bind plus affinity gel matrix (GammaBind Plus Sepharose; GE Healthcare, Uppsala, Sweden). Antibodies were eluted using 0.1 M glycine (pH 2.7) and neutralized with one-eighth volume of 1 M Tris–HCl (pH 9.0). The peak fractions were dialyzed three times against PBS (pH 7.0) extensively. Monoclonal antibodies were quantified spectrophotometrically at 280 nm. The antibody solutions were filter-sterilized using 0.2 µm syringe filters (Dynagard; Spectrum Laboratories, CA, USA) and stored at 4°C until used. Endotoxin content in the antibody solutions was found to be below the detection limit (less than 0.1 EU/mg) as analyzed using Limulus amebocyte lysate assay kit (Limulus Amebocyte Lysate, Lonza, USA).

### Enzyme-Linked Immunosorbent Assays

Several monoclonal antibodies were generated from bCXI-immunized mice and their binding specificity was determined. Whole blood was collected from the mouse retro-orbital venous sinus on day 21 and day 70 after immunization with CII. 32 sera samples in total from each time point were measured for the anti-CII and anti-CXI antibody responses. 96-well flat-bottom ELISA plates (Nunc MaxiSorp; Denmark) were coated overnight with 5 µg/ml of purified rCXI, rCII, and bCXI, either native or denatured, or with 5 µg/ml synthetic peptides in PBS at 4°C. After the blockage with 3% non-fat milk in PBS at room temperature (RT) for 2 h, purified antibodies diluted according to a previously determined concentration and 1:1,000 diluted serum samples were incubated at RT for 2 h. The antibody titers were evaluated using HRP-conjugated anti-kappa-specific antibody (1:4,000, Southern Biotech) and ABTS (Roche) as detect system. For isotype specific assessment, biotinylated goat anti-mouse-IgM, -IgG1, -IgG2a, -IgG2b, or -IgG3 reagents (Southern Biotech) were used.

### IL-2 Cytokine ELISA

96-well flat-bottom ELISA plates were coated overnight with 2 µg/ml of IL-2 antibody (Jes6-1A12) in PBS at 4°C. After the incubation of supernatant from T cell recall assay for 2 h at RT, the biotinylated IL-2 antibody (Jes6-5H4) was incubated for 1 h at RT. IL-2 titers were detected using europium-labeled streptavidin (DELFIA, 1:1,000 in assay buffer) on a Synergy 2 multi-mode plate reader (BioTek).

### Bead-Based Multiplex Immunoassays

Autoantibody responses were analyzed by using the Luminex technology as described previously (28). Briefly, all the biotinylated peptides were captured on beads coated with NeutrAvidin (Thermo Fischer Scientific). Human serum samples were diluted 1:100 (v/v) and purified antibodies were prepared into a final concentration of 1 µg/ml in assay buffer (3% BSA, 5% milk powder, 0.1% ProClin300, 0.05% Tween 20, 100 µg/ml NeutrAvidin in PBS) and incubated for 1 h at RT on a shaker. Then, the samples were either transferred to a 384-well plate (Greiner Bio-One) containing the peptide-coated beads by a liquid handler (CyBi-SELMA, CyBio) or to a 96-well plate (Greiner Bio-One) by manual pipetting. After incubation at RT on a shaker for 75 min, all the beads were washed with 0.05% Tween-20 in PBS (PBST) on a plate washer (EL406, Biotek or Bioplex Pro Wash station, Biorad), and then resuspended in a solution containing the secondary anti-human, anti-mouse, or anti-rat IgG Fcγ-PE (Jackson Immuno Research). After 40 min of incubation, the beads were washed with PBST and the fluorescence intensity was measured using FlexMap3D or Luminex 200 (Luminex Corp.) instrument. The median fluorescence intensity (MFI) was used to quantify the interactions of the antibody with the given peptides. For the comparison of responses to peptides in human and rat samples, the ratio value, calculated by dividing the MFI value for the peptide of interest by the median MFI value of five cyclic control peptides, was used.

### Patients

In the present study, samples from a subset of previously described Epidemiological Investigation of RA (EIRA) cohort (29), consisting of 1,984 RA patients, included at disease onset and 400 age and sex-matched healthy controls, were used. RA was defined according to the American College of Rheumatology (ACR) 1987 criteria (30). This study was carried out in accordance with the recommendations of EIRA 96-174, EIRA II 2006/476-31/4, and 2007/718-32 guidelines with written informed consent from all subjects. The protocol was approved by the ethics committee at the Karolinska Institute and by Regional Stockholm ethics committee.

### Induction and Evaluation of Arthritis (CIA, PIA, CAIA)

Collagen II-induced arthritis (C^II^IA) or collagen XI-induced arthritis (C^XI^IA) was induced by an intradermal injection of 100 µg of rCII or rCXI emulsified in an equal volume of CFA in DBA/1J mice, respectively. Followed with a booster, immunization of 50 µg of rCII or rCXI emulsified in IFA on day 21. 100 µg rCII emulsified in an equal volume of IFA or 100 µl of Pristane (2,6,10,14 tetramethylpentadecane, 95%, Acros Organics, Morris Plains, NJ, USA) was injected intradermally to induce C^II^IA or PIA (21, 31) in 8–12 weeks old DA rats.

The J1 epitope (MPGERGAAGIAGPK)-specific antibody M2139 used in this study was produced as described earlier (32). The cocktail of two monoclonal antibodies was prepared by mixing equal concentrations of each of the sterile filtered antibody solution to achieve a final amount of 9 mg. Mice were injected with either 9 mg of M2139 + L10D9, M2139 + L5F3 or L10D9, or 4.5 mg of M2139 antibodies intravenously. All the mice received (25 μg/mouse) lipopolysaccharide from *Escherichia coli* O55: B5 (Sigma-Aldrich, Saint Louis, MO, USA) intraperitoneally on day 5 to enhance the disease incidence and severity.

Mice and rats were examined for arthritis development with the identity of the animals blinded for the investigator using an extended scoring protocol. Briefly, clinical arthritis is defined as swelling and redness in the joint and was scored as below: 1 point for each inflamed toe or knuckle, 5 points for an inflamed wrist or ankle, resulting in a maximum of 15 points per limb and a maximum of 60 points per animal (33).

### Histology

To investigate the antibody binding with joints *in vivo* and *in vitro*, limbs from 2 days old neonatal BQ.Cia9i mice injected intraperitoneally with 100 µg biotinylated M2139, L10D9, L5F3, L7D8 antibodies, or PBS were collected and snap frozen, cryo-sectioned, whereas the paws from adult healthy or chronic C^II^IA mice were harvested, fixed, decalcified, dehydrated, paraffin-embedded, and 7 µm thick sections were used. The sections from biotinylated antibodies injected mice were fixed in 4% paraformaldehyde for 5 min. The sections from naïve neonatal mice, and from paraffin-embedded joints, which underwent antigen retrieval, were subjected to 5 µg/ml biotinylated M2139, L10D9, L5F3, L7D8 antibodies, or PBS for 40 min in RT. After blocking with 3% H_2_O_2_ for 10 min, 5% BSA + 2% rat sera for 30 min, streptavidin for 15 min, biotin for 15 min, all the sections were incubated with ExtrAvidin-Peroxidase solution (Sigma-Aldrich, Saint Louis, MO, USA) for 30 min and developed with diaminobenzidine (DAB Kit; Dako, Copenhagen, Denmark) for 8–9 min (sections from biotinylated antibodies injected mice) or 3 min (sections from naïve neonatal, adult healthy, and chronic C^II^IA mice).

For histological assessments, paws from the BQ.Cia9i and B10RIII mice used for CAIA experiments were dissected, fixed, decalcified, dehydrated, and then paraffin-embedded. Sections (7 µm) were stained with hematoxylin/eosin to observe joint morphology.

### Immunofluorescence

Six-week-old BQ.Cia9i mice were injected with 1 mg of biotinylated M2139, L10D9, L5F3, L7D8 antibodies, or PBS intravenously. After 24 h, the nose was harvested, snap frozen, and kept in −80°C. Transversal snout sections (7 µm) were fixed in acetone on ice for 10 min, dried for 10 min, and hydrated in 1× PBS for 15 min. After blocking with 1× PBS containing 2% BSA + 0.1% Tween-20 for 45 min, the sections were incubated with streptavidin-conjugated Alexa Fluor 568 (Invitrogen, S11226, 1:300 diluted) for 60 min in RT and mounted using VECTASHIELD^®^ Mounting Medium with DAPI (Vector, H-1200). The slides were dried for 15–30 min before scanning under a confocal microscope.

### Statistical Analyses

Quantitative data are expressed as a mean ± SEM. Arthritis incidence was analyzed using Fisher’s exact test, whereas the comparison of arthritis severity and serum antibody response between groups were performed using the non-parametric Mann–Whitney *U*-test. Pearson correlation test was used for analyzing the correlation of antibody response to arthritis disease severity. *p*-Values less than 0.05 were considered statistically significant.

## Results

### Serum Antibody Response to CII and CXI in Mice

**Figure 1:**
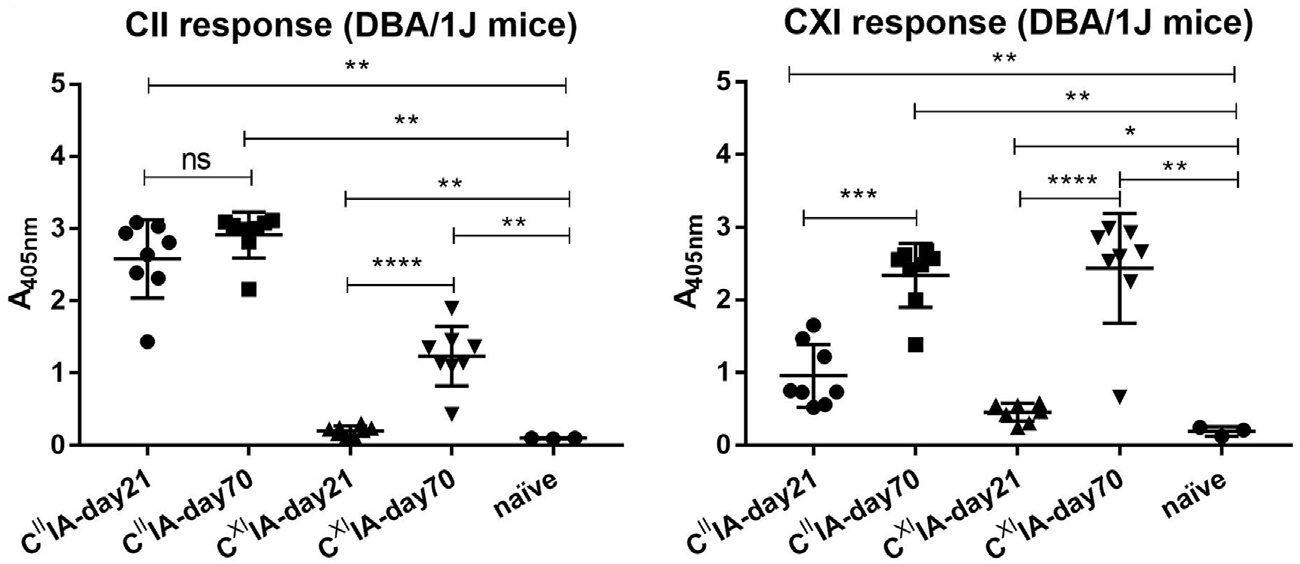
Serum antibody response to native CII and CXI in C^II^IA and C^XI^IA mice. Serum antibody response to native CII (left panel) and CXI (right panel) protein on day 21 or day 70 were measured in C^II^IA and C^XI^IA DBA/1J mice using enzyme-linked immunosorbent assays (ELISA). Naïve mice sera samples were served as negative control. Mann–Whitney *U*-test was used for group comparison.

In order to understand the potential relationships between serum antibody profiles and arthritis development in C^II^IA and C^XI^IA, we immunized the A^q^-expressing DBA/1J mice with rCII or rCXI. Sera were collected on days 21 and 70. We observed severe arthritis with high frequency in C^II^IA mice but only very mild disease with low frequency in C^XI^IA mice (Table 1). Elevated serum antibody responses to CII and CXI were observed throughout the disease course in both C^II^IA and C^XI^IA mice, and the responses were increasing with time. Anti-CXI antibody response in C^II^IA mice was higher than that in C^XI^IA mice in the early stage (day 21: *p* = 0.0009), but not in the late stage (day 70: *p* = 0.1393) (Figure 1). No correlation could be observed between levels of the anti-CII/CXI antibody responses and disease severity (data not shown).

**Table 1:** Disease incidence and Max arthritis score of C^II^IA and C^XI^IA mice.

Immunogen	Strain	Gender	Age (weeks)	Incidence	Max arthritis score (mean ± SEM)
CII/CFA	DBA/1J	Male	10	7/8	20.13 ± 3.451
CXI/CFA	DBA/1J	Male	10	1/8	0.25 ± 0.25

*C^II^IA, collagen II-induced arthritis; C^XI^IA, collagen XI-induced arthritis; CII, collagen II; CXI, collagen XI; CFA, complete Freund’s adjuvant; IFA, incomplete Freund’s adjuvant*.

### CXI Harbors the Same T Cell Glycosylated Epitope (Glycosylation at Position 264) as CII

**Figure 2:**
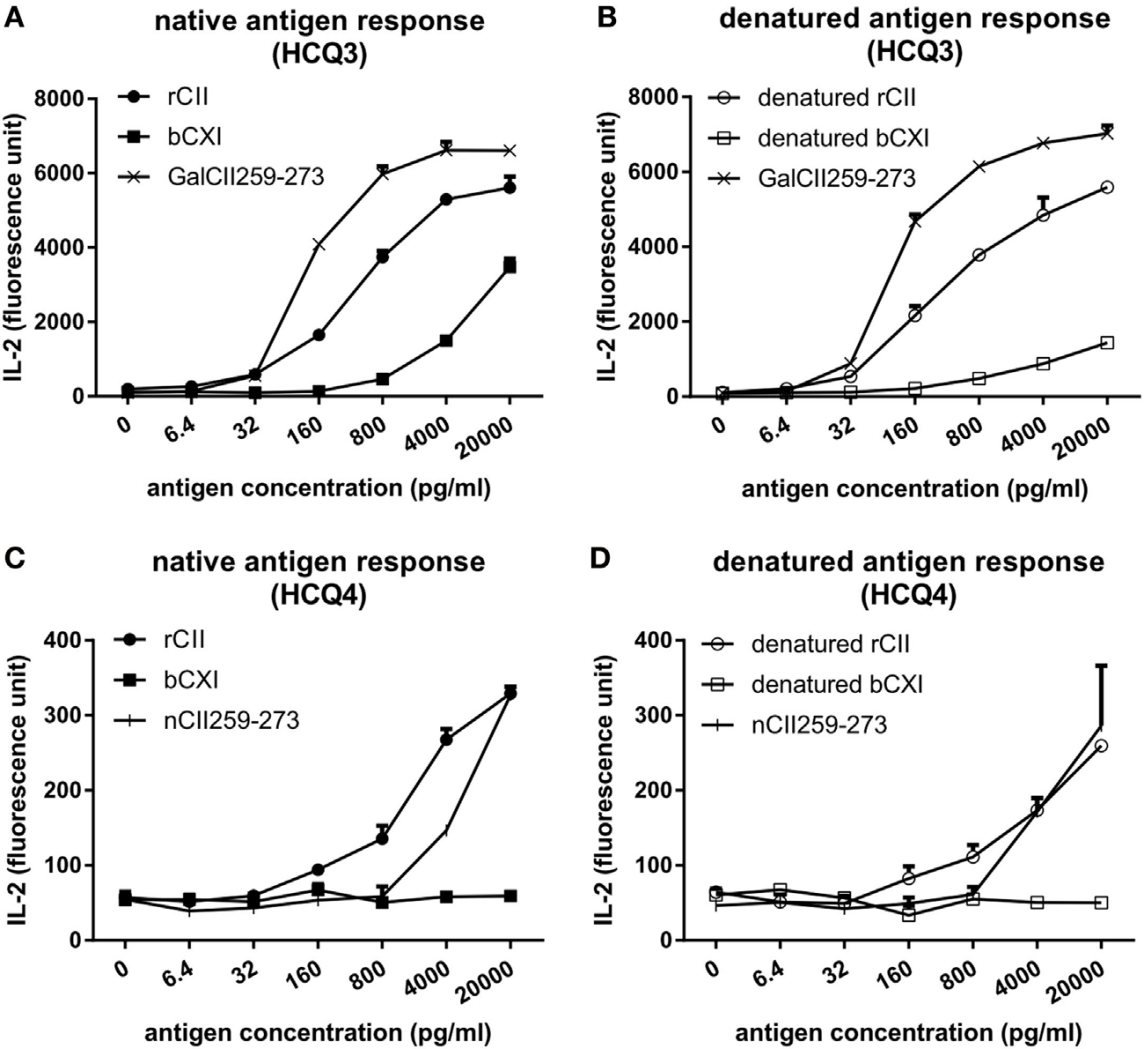
T cell recall assay for CII and CXI. The response of HCQ3 and HCQ4 hybridoma to native **(A,C)** and denatured **(B,D)** collagens. The supernatant was collected and the IL-2 secretion was measured by cytokine enzyme-linked immunosorbent assay. Glycosylated CII259-273 peptide (GalCII259-273) and naked CII259-273 peptide (nCII259-273) were served as positive control peptides for HCQ3 and HCQ4 hybridoma, respectively. rCII, rat Collagen II; bCXI, bovine collagen XI.

To investigate whether CII and CXI harbor the same T cell epitope, T cell hybridomas HCQ3 and HCQ4, which are specific for glycosylated CII259-273 (GalCII259-273, glycosylation at position 264) and naked CII259-273 (nCII259-273, lysine at position 264) peptide, respectively, were used for T cell recall assay. HCQ3 showed a response to both native and denatured bCXI, but at a lower level compared to that of rCII and denatured rCII (Figures 2A,B). HCQ4 did not react to either native or denatured bCXI (Figures 2C,D). These data showed that CXI harbors the same posttranslationally modified epitope, which has glycosylation at position 264 as that of CII. The sequence alignment for T cell epitope between two different species was shown (Figure S2 in Supplementary Material).

### Generation and Characterization of Anti-CXI Monoclonal Antibodies

**Figure 3:**
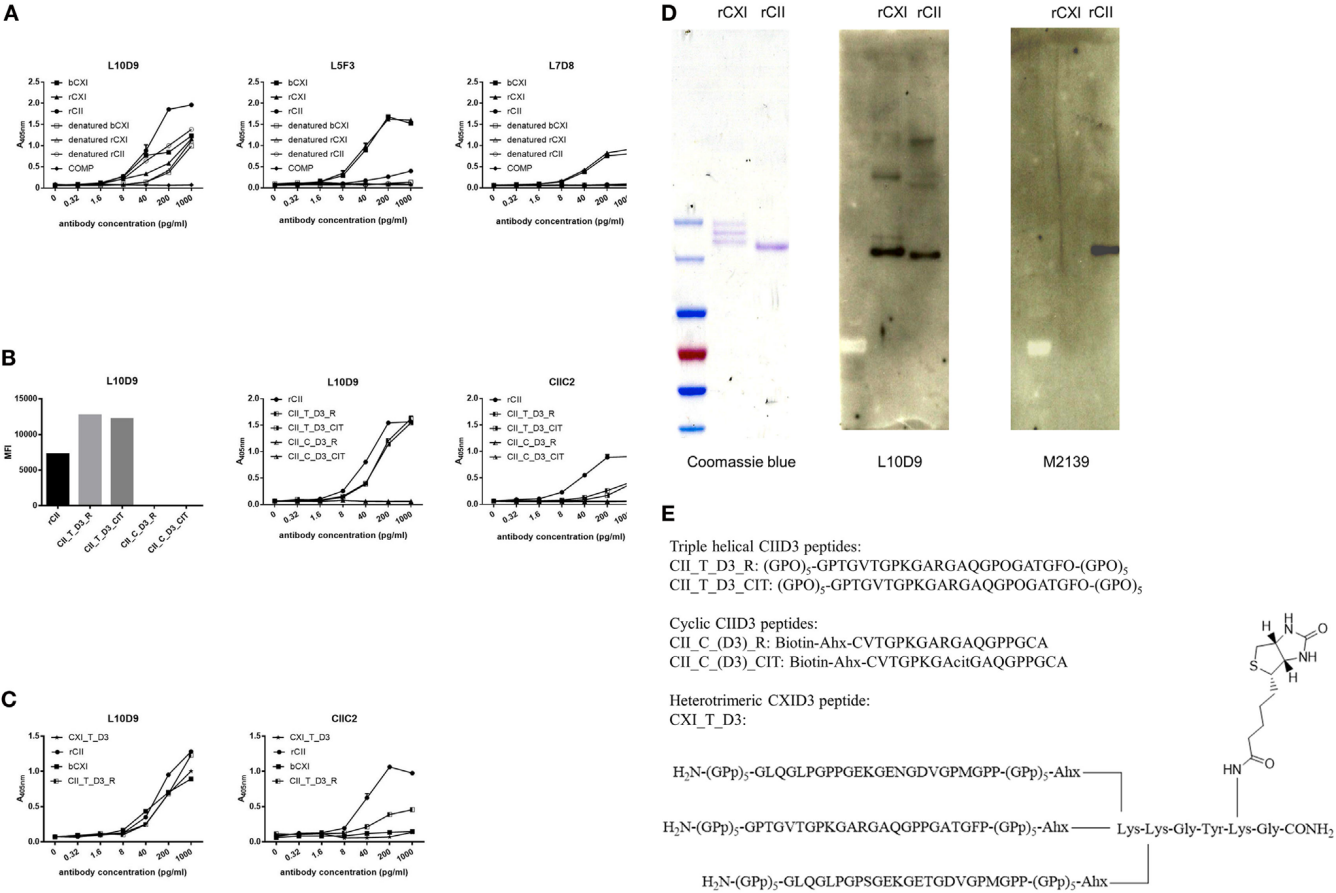
L10D9 binds to CXI, cross-react with CII due to sequence similarity. **(A)** Kinetics of L10D9, L5F3, and L7D8 antibody response to bCXI, rCXI, rCII, and their denatured forms. Cartilage oligo matrix protein (COMP) was served as a negative control. Bead-based multiplex immunoassays [**(B)**, left panel] and enzyme-linked immunosorbent assays (ELISA) [**(B)**, middle and right panel] were used for the epitope mapping of L10D9. **(C)** The binding of L10D9 and CIIC2 to the heterotrimeric CXI_T_D3 peptide. **(D)** SDS-PAGE for rCXI and rCII (left panel). Western blotting for L10D9 (middle panel) and M2139 (right panel). **(E)** Sequence of the peptides involved in ELISA and bead-based multiplex immunoassays.

A series of 19 anti-CXI monoclonal antibodies were generated from DBA/1J mice immunized with bCXI protein. Antibodies with different isotypes and affinities to rCII, rCXI and bCXI were shown in the table (Table 2). L10D9 showed a strong binding to rCII, rCXI, bCXI, and their denatured forms, as revealed by ELISA (Figure 3A). L5F3 and L7D8 bound strongly to native rCXI and bCXI, but not their denatured forms. In contrast, a weak (L5F3) or no (L7D8) binding to CII was observed, indicating that these two clones bound specifically to CXI (Figure 3A).

**Table 2:** The characteristics of 19 monoclonal CXI antibodies.

Clones	Isotype	bCXI	rCXI	rCII	Bind cartilage
L3C11	IgG1	+	+	+	P
L5F3	IgG2b	++	++	+	P
L10D5	IgG2b	++	++	+	P
L10D9	IgG2a	+++	+++	++++	P
L13G8	IgG2b	++	++	+	P
L1D7	IgG2b	++	−	−	N
L1D8	IgG2b	++	++	−	N
L2D9	IgG2a, 2b	+	−	−	N
L2G11	IgG2b	−	+	−	N
L3E2	IgG2b	+	+	−	N
L7A2	IgG2b	+	+	−	N
L7B6	IgG1	++	++	+	N
L7D8	IgG2a	++	++	+	N
L7H5	IgG2b	+++	+	−	N
L8F2	IgG1	++	++	+	N
L10B9	IgG2a	+++	−	−	N
L11C5	IgG1	+++	+++	+	N
L14E2	IgG1	+	+	−	N
L16G4	IgG1	+++	−	+	N

*Scale for optical density (OD) values: − denotes <3 times SEM of negative control; + denotes >mean + 3SD of negative control; ++ denotes >0.5 OD; +++ denotes >1.0 OD; ++++ denotes >1.5 OD. P, positive binding to the cartilage; N, negative binding to the cartilage; rCXI, rat CXI*.

To identify the potential binding of CXI antibodies to cross-reactive epitopes between α3(XI) and α1(II), we performed bead-based multiplex immunoassay using 198 triple helical CII peptides, including those with citrulline modified versions. L10D9 showed a unique binding to the triple helical peptides covering the D3 epitope of CII (34), both unmodified and modified forms, but neither the corresponding cyclic peptides covering the same region nor other CII peptides (Figure 3B) showed reactivity. The same specificity could also be confirmed using ELISA (Figure 3B). CIIC2, an antibody specific for the D3 epitope of CII, showed positive binding (Figure 3B). No such binding could be found for L5F3, L7D8, and other CXI specific antibodies in our current CII peptides library (data not shown).

In order to study which α chains of CXI are needed for the binding of the L10D9 antibody to the antigens, a heterotrimeric CXI_T_D3 peptide of 24 amino acid in length was synthesized. L10D9 bound specifically to this peptide, which indicated that only the α chain shared between CXI and CII [α3(XI)/α1(II)] was needed for binding of this antibody. The CII-specific CIIC2 antibody failed to bind to the CXI_T_D3 peptide indicating that more than one α chain from CII was needed for binding of this antibody (Figure 3C). The binding of L10D9 to α3(XI)/α1(II) was further confirmed by SDS-PAGE and Western blot. L10D9 showed unique binding to α3(XI) and α1(II), while the CII specific control antibody M2139 bound only to α1(II) (Figure 3D). L10D9, a CXI-responding antibody, cross-reacts to CII due to sequence identity (GPTGVTGPKGARGAQGPOGATGFO). The CIID3 and CXID3 peptide sequences and the sequence alignments among four different species are shown in Figure 3E and Figure S2 in Supplementary Material.

### D3 Epitope-Specific Antibody Binds Cartilage, While CXI Specific Antibodies Showed Negligible or Very Weak Binding

**Figure 4:**
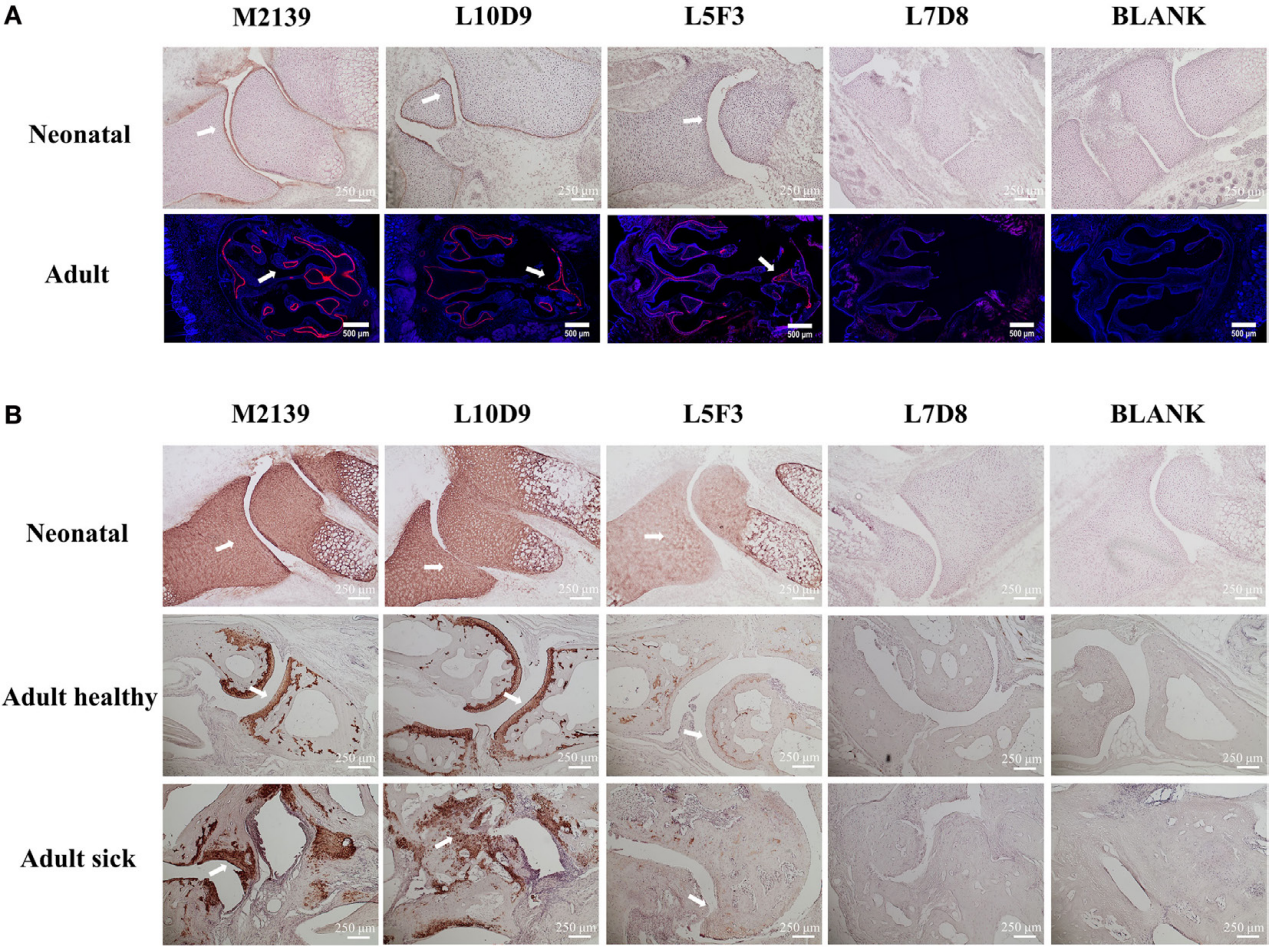
L10D9 binds to cartilage both *in vivo* and *in vitro*. *In vivo* **(A)** and *in vitro* **(B)** histology staining of joint or transversal snout sections are shown. Positive binding to cartilage surfaces are marked by arrows.

To test whether L10D9, L5F3, and L7D8 antibodies could bind to neonatal and adult cartilage *in vivo*, these antibodies were biotinylated and then injected intraperitoneally into neonatal or 6 weeks old BQ.Cia9i mice. The biotinylated M2139 antibody was used as a positive control. Joint tissues from the neonatal and snout tissues from the adult mice were obtained 24 h later after injection, and tissues were analyzed for antibody binding by immunohistochemistry (due to the difficulty of the sectioning for adult joints, we took snout tissue). The L10D9 antibody showed a similar staining pattern as the anti-CII antibody M2139, with specific binding along neonatal cartilage surface. L5F3 and L7D8 antibodies showed no clear and specific staining (Figure 4A, upper panel). In the adult tissue, the L10D9 antibody demonstrated strong staining along the cartilage surface. The CXI-specific antibody L5F3 showed a similar but weaker staining as compared to the anti-CII antibody, whereas the binding of the L7D8 antibody to the adult tissue was not detected (Figure 4A, bottom panel).

For the *in vitro* binding capacity of the L10D9 antibody to cartilage, paw sections from naïve neonatal BQ.Cia9i, adult mice with or without arthritis were incubated with biotinylated M2139, L10D9, L5F3, or L7D8 antibodies, followed by detection of antibodies binding to the sections. Both L10D9 and L5F3 antibodies showed clear cartilage staining on the tissue sections from neonatal as well as adult healthy and sick mice, whereas L7D8 antibody binding was negative (Figure 4B).

### L10D9 Induces and Enhances Acute Arthritis in Naïve Mice, but Not the CXI-Specific Antibody

**Figure 5:**
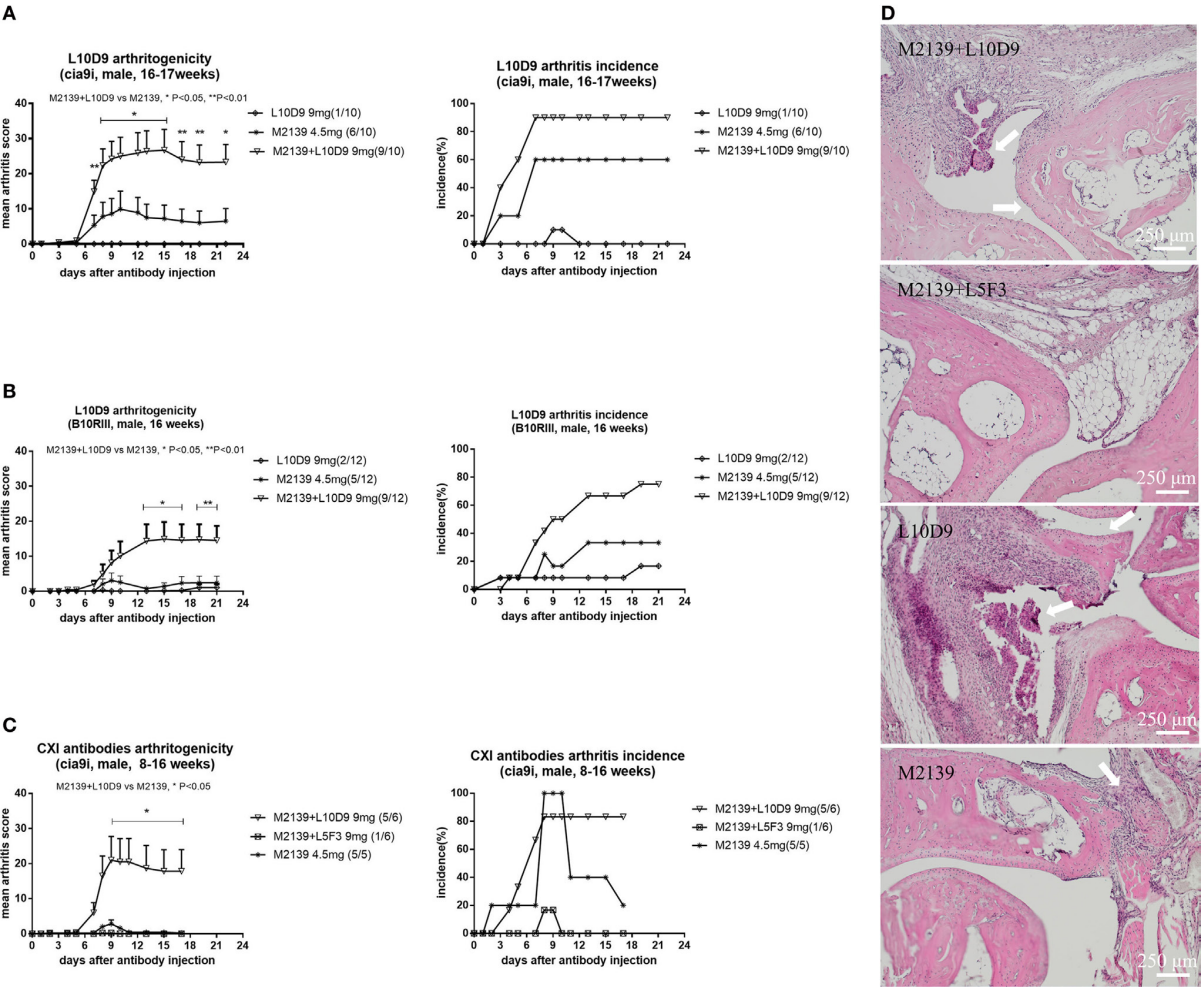
L10D9 mediates arthritis in mice. Mean arthritis score and arthritis incidence in B10RIII strain **(B)** and BQ.Cia9i strain **(A,C)** are indicated. **(D)** Histology of joint sections. Results shown are representative pictures of each group. Infiltrating cells, glycosaminoglycan loss, and joint surface erosions are indicated with arrows. Statistics were determined by the Mann–Whitney *U*-test for daily mean arthritis score and Fisher’s exact test for the disease incidence between groups.

To investigate the arthritogenicity of the antibodies, 8–17 weeks old BQ.Cia9i male mice were injected with 4.5 mg of M2139, 9 mg of L10D9, M2139 + L5F3, or M2139 + L10D9 antibody intravenously. A single injection of M2139 or L10D9 antibody induced very mild arthritis, while M2139 antibody combined with L10D9 antibody developed more severe disease compared to M2139 group (*p* < 0.05) (Figure 5A). The same finding is also seen in the B10RIII strain, suggesting that L10D9 has the capacity to induce mild arthritis by itself and a strong enhancing arthritis effect when combined together with the M2139 antibody (Figure 5B). No arthritis enhancing effect can be found for L5F3 antibody (Figure 5C). Representative histology pictures from each group show the infiltrating cells, glycosaminoglycan loss, and joint surface erosions (Figure 5D).

### Elevated Antibody Response to the D3 Epitope in RA Patients and Arthritic Rodents

**Figure 6:**
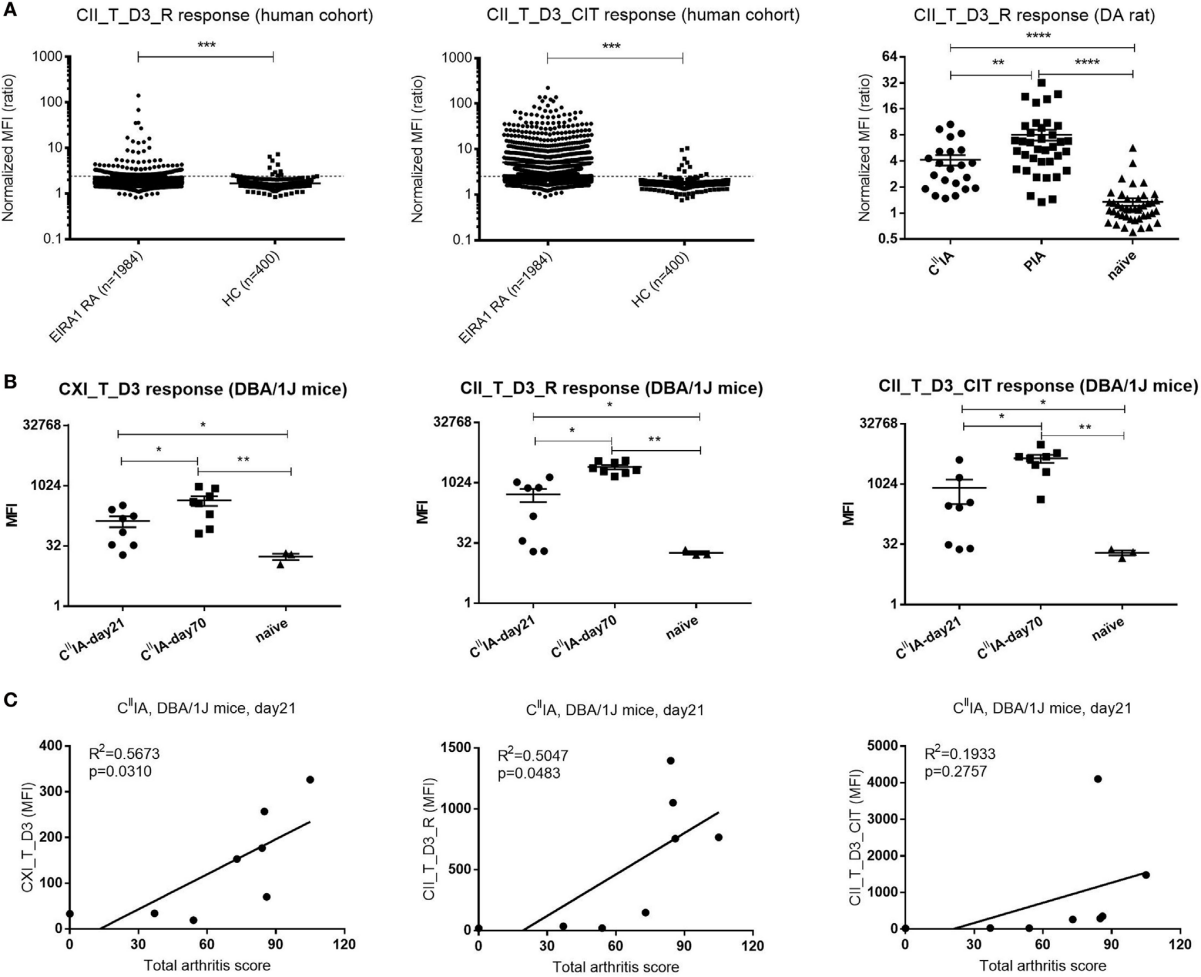
The D3 epitope response in Epidemiological Investigation of RA (EIRA) cohort and arthritic rodents, and the correlation between D3 epitope response and arthritis severity in C^II^IA mice. **(A)** IgG response of the (EIRA) cohort (rheumatoid arthritis: *n* = 1,984, HC: *n* = 142) to CII_T_D3_R and CII_T_D3_CIT (left and middle panel). The broken line indicates the cutoff (median + 5 × MAD) based on healthy controls. Anti-collagen antibody level against CII_T_D3_R peptide in C^II^IA (*n* = 23), Pristane-induced arthritis model (*n* = 37), and naïve (*n* = 44) DA rats (right panel). Data are shown as normalized mean fluorescence intensity (MFI) ± SEM. **(B)** Anti-collagen antibody against CXI_T_D3, CII_T_D3_R and CII_T_D3_CIT in C^II^IA DBA/1J mice. **(C)** Correlation between serum antibody response to CXI_T_D3, CII_T_D3_R, and CII_T_D3_CIT and total arthritis score in C^II^IA mice. Statistics were determined by the Mann–Whitney *U*-test and Pearson correlation test for group comparison and correlation analysis, respectively.

To investigate the value of D3 epitope response in a clinical setting and to serologically characterize the experimental rodent models; sera from humans, rats, and mice were measured for the response toward D3 epitope. We found that serum antibody response to D3 epitope was significantly elevated in RA patients’ sera in the EIRA cohort compared to healthy controls (*p* < 0.0001). In particular, strong responses were found toward the citrullinated D3 variant (CII_T_D3_CIT) (Figure 6A, left and middle panel). We next investigated whether similar responses can be detected in murine models of arthritis. In rats, at day 23 after immunization with CII/IFA or Pristane, the anti-CII antibody response was most pronounced in those injected with Pristane followed by CII-immunized animals and, both types of immunization mounted a CII_T_D3 specific responses that were significantly increased compared to naïve rats (Figure 6A, right panel). Similar findings were observed in C^II^IA mice. Anti-D3 epitope response against CXI_T_D3, CII_T_D3_R (arginine version), and CII_T_D3_CIT (citrulline version) peptides can be found throughout the disease course, which increased as the disease progressed with time (Figure 6B).

To understand the value of the anti-D3 antibody response in disease development, we correlated D3 epitope specific antibody response to total arthritis score in C^II^IA mice. Both the antibody response to CXI_T_D3 and to CII_T_D3_R was positively correlated with disease severity, but not the anti-CII_T_D3_CIT response (Figure 6C). We also observed increased antibody response to other major CII epitopes and the positive correlation between the antibody response to the C1 epitope and arthritis severity in C^II^IA mice (Figures S1A,B in Supplementary Material).

## Discussion

Antibodies targeting cartilage is likely an important step toward arthritis development in both humans and experimental animals. Here, we have identified the structural requirements for an antibody mediated cross-reactive response to CII and CXI, which could explain why this response is highly arthritogenic. Collagen fibers in normal articular cartilage are mainly composed of CII together with the minor components CIX and CXI, in which CXI molecules are intermingled in the cartilage collagen fibers. CXI and CII molecules have similar triple helix structure, with three identical α chains for CII and three different α chains for CXI, in which α3(XI) is coded from the same gene as α1(II). Several studies indicate the potential arthritogenic and immunogenic effects of CXI in mice (16, 17). In our current study, we could only observe very mild disease with a low incidence of arthritis after immunization with CXI in mice, while in parallel, the C^II^IA mice developed severe arthritis with high incidence. Elevated serum antibody response against CII and CXI could be found throughout the disease process in both C^II^IA and C^XI^IA mice, which increased with time. Both serum CII and CXI antibody responses were significantly higher in C^II^IA compared to C^XI^IA mice, except the anti-CXI antibody responses during the late stage of the disease. One possible explanation for the cross-reactive responses could be the common α chain between CII and CXI, the other could be due to the destruction of cartilage and subsequent exposure of CXI molecules during arthritis development. It is well known that injection of antibodies specific for CII after binding to cartilage *in vivo* can induce arthritis in rodents (32). High level of antibodies against CII could be the reason why C^II^IA mice developed severe disease compared to C^XI^IA mice, which had a much lower level of CII antibody titer. Although there was a high titer of CXI specific antibodies existing in C^XI^IA mice, those antibodies could not contribute to the disease because the CXI molecules are not exposed for binding of antibodies *in vivo*. A perquisite for a strong immune response to CXI is that T cell could be specifically activated, and here, we could show that native CXI harbored the same glycosylated T cell epitope (GalCII259-273, glycosylation at position 264) as CII. Though the response to CXI by Gal-264-specific T cell hybridoma is significantly lower than to CII, this could be explained by the amount of the identical alpha chain containing the T cell epitope, as CII is a homotrimer whereas CXI only have one chain (α3) with the epitope. However, it can still be the difference in glycosylation pattern, in particular, with other forms of the lysine 264 side chain or glycosylation at the lysine 270 side chain, which is not addressed here. However, Gal-264, the most important glycosylation form for activation of T cells are present on CXI. The sequence similarity and shared T cell epitope is a prerequisite for the occurrence of high-affinity IgG cross-reactive antibodies after CII or CXI protein immunization.

We believe our most interesting finding is that we could isolate a CXI/CII cross-reactive autoantibody L10D9, from mice immunized with CXI, which could contribute to the development of arthritis. The L10D9 needed only one common α chain for successful binding, and it is possible that the alpha chains are partially flexed out from the triple helical structure in order to be accessible. This finding was further confirmed by Western blotting, showing that L10D9 only bound to the α3 chain of CXI and α1 chain of CII. The identified epitope is localized to an earlier defined CII epitope, denoted as D3. The L10D9 antibody also bound successfully to the synthesized heterotrimeric CXI_T_D3 peptide in which α3(XI) has the same sequence to α1(II) but not to the linear peptide with the same sequence as the CII_T_D3 triple helix peptide (data not shown). Thus, the epitope is most likely exposed by a structure formed by a partially unwound alpha chain from the triple helical structure. The L10D9 antibody bound as strongly as CII antibody to cartilage *in vivo* and *in vitro* whereas the CXI specific antibodies showed either much weaker or negligible staining. The weak staining could possibly be explained by the low level of cross-reactivity of L5F3 antibody to CII (see the ELISA result), and the absence of positive staining for the highly specific CXI antibody L7D8 further confirms that CXI molecules are not exposed in the joint tissue for binding. This likely also explains that the CXI specific antibodies could not induce arthritis. The situation could, however, be different in affected joints as it is then possible that more CXI molecules are exposed and accessible for both inducing not only a specific B cell response but also for targeting with antibodies at a later stage.

Importantly the L10D9 antibody is highly arthritogenic and is among the few antibodies that can actually induce arthritis by itself. Induction of arthritis by antibodies is clearly much more efficient if exposed to polyclonal or oligoclonal antibodies, but a few monoclonal antibodies seem to have the property to induce arthritis by themselves. Examples of these include the anti-CII antibody M2139, used in this study, the anti-citrulline protein antibody ACC1 that cross-reacts to CII (35) and the CXI-CII cross-reactive antibody L10D9 described in the current study. All of these antibodies seem to have the possibility to target a single alpha chain within a triple helical structure, i.e., targeting a structure formed by a dynamically flexed out alpha chain from an unstable triple helical molecule and with the capacity to strongly bind to cartilage *in vivo*.

Furthermore, we detected antibodies targeting CII, CXI, or the triple helical D3 epitope during the development of arthritis in both experimental animal models and in RA patients. It is likely that such antibodies play a pathogenic role and could further perpetuate the disease during the chronic relapsing state of the disease.

## Ethics Statement

The human study was carried out in accordance with the recommendations of EIRA 96-174, EIRA II 2006/476-31/4, and 2007/718-32 guidelines with written informed consent from all subjects. The protocol was approved by the ethics committee at the Karolinska Institutet and by Regional Stockholm ethics committee. The animal study was carried out in accordance with the recommendations of N490/12, N35/16 (Stockholm ethical committee) and 86/609/EEC guidelines (European Community Council Directive). The protocols were approved by local animal welfare authorities.

## Author Contributions

DT: designed the study, performed most of the experiments, data analysis, interpreted the data, and wrote the first draft of the manuscript. EL, BL, and AY: performed experiments, helped with data analysis interpretation, and manuscript writing. KN, CG, and IG: helped with experimental design and corrected manuscript. JV and JK: helped with triple helical peptide synthesis and analysis and corrected manuscript. LL, AC, and MB: helped with confocal analysis and corrected manuscript. LK: provided human cohort. RH: designed the study, data interpretation, manuscript writing, and supervised the project. All authors approved the final manuscript.

## Conflict of Interest Statement

The authors declare that the research was conducted in the absence of any commercial or financial relationships that could be construed as a potential conflict of interest.
