# Supplementary material for: A Shared Epitope of Collagen Type XI and Type II Is Recognized by Pathogenic Antibodies in Mice and Humans with Arthritis
Source: Front Immunol. 2018 Apr 12;9:451. doi: 10.3389/fimmu.2018.00451 (PMC5906551; doi:10.3389/fimmu.2018.00451)
Supplement: Supplementary file 1 [file data_sheet_1.docx]

Supplementary Material

A Shared Epitope of Collagen Type XI and Type II is Recognized by Pathogenic Antibodies in Mice and in Humans with Arthritis

Dongmei Tong^1,2,3^, Erik Lönnblom^1^, Anthony C. Y. Yau^1^, Kutty Selva Nandakumar^1,3^, Bibo Liang^1,2^, Changrong Ge^1^, Johan Viljanen^4^, Lei Li^5^, Mirela Bãlan^6^, Lars Klareskog^7^, Andrei S. Chagin^5,8^, Inger Gjertsson^9^, Jan Kihlberg^4^, Ming Zhao^2^*, and Rikard Holmdahl^1,3^*

^1^Section for Medical Inflammation Research, Department of Medical Biochemistry and Biophysics, Karolinska Institute, Stockholm, Sweden.

^2^Department of Pathophysiology, Key Lab for Shock and Microcirculation Research of Guangdong, Southern Medical University, Guangzhou, China.

^3^Medical Immunopharmacology Research, School of Pharmaceutical Sciences, Southern Medical University, Guangzhou, China

^4^Section of Organic Chemistry, Department of Chemistry - Biomedical center, Uppsala University, Uppsala, Sweden.

^5^Department of Physiology and Pharmacology, Karolinska Institute, Stockholm, Sweden.

^6^Section of Vascular Biology, Department of Medical Biochemistry and Biophysics, Karolinska Institute, Stockholm, Sweden.

^7^Rheumatology Unit, Department of Medicine, Karolinska Institute and Karolinska University Hospital, Stockholm, Sweden

^8^Institute for Regenerative Medicine, Sechenov First Moscow State Medical University, Moscow, Russian Federation

^9^Department of Rheumatology and Inflammation Research, University of Gothenburg, Gothenburg, Sweden

* Shared corresponding authors

**Correspondence**:

Prof. Rikard Holmdahl: rikard.holmdahl@ki.se

Prof. Ming Zhao: 15602239057@163.com


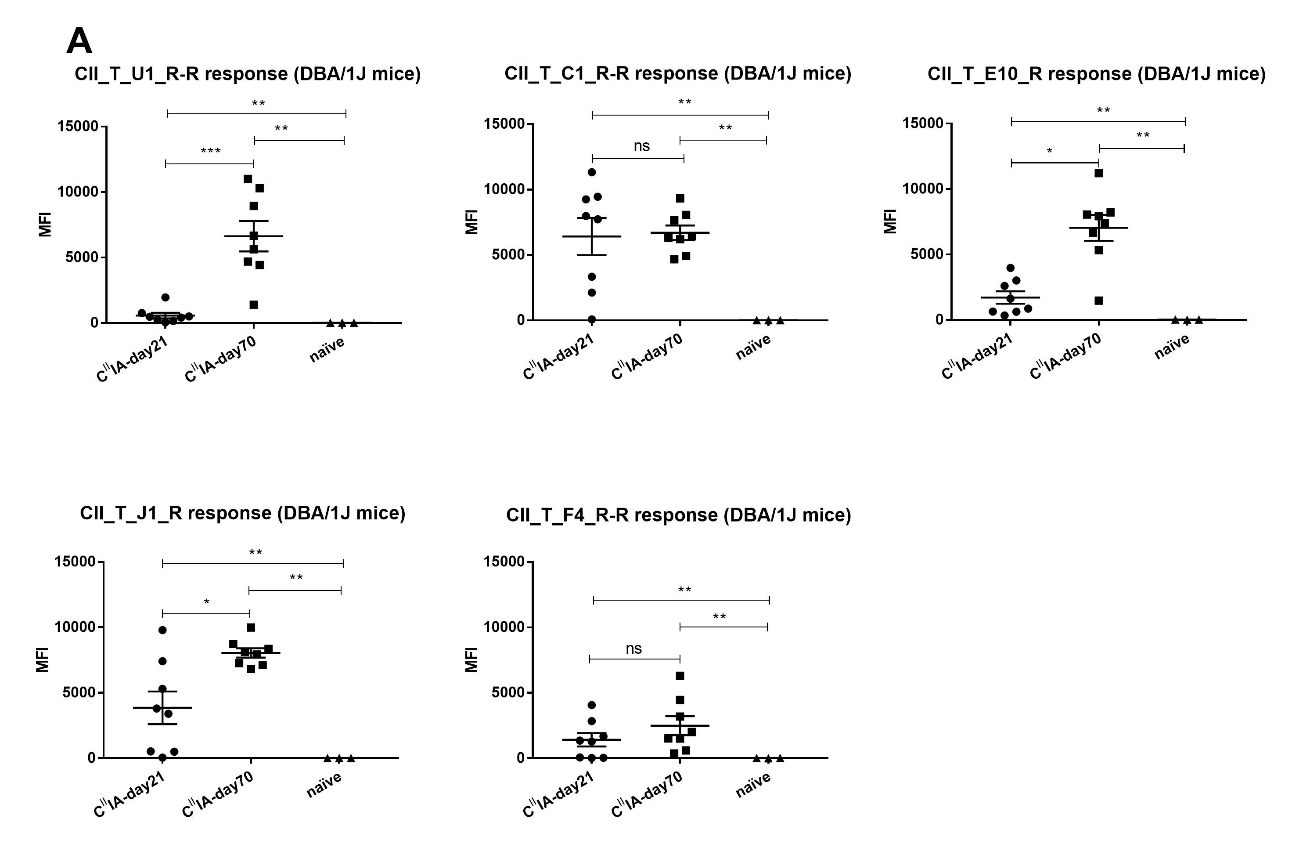


**
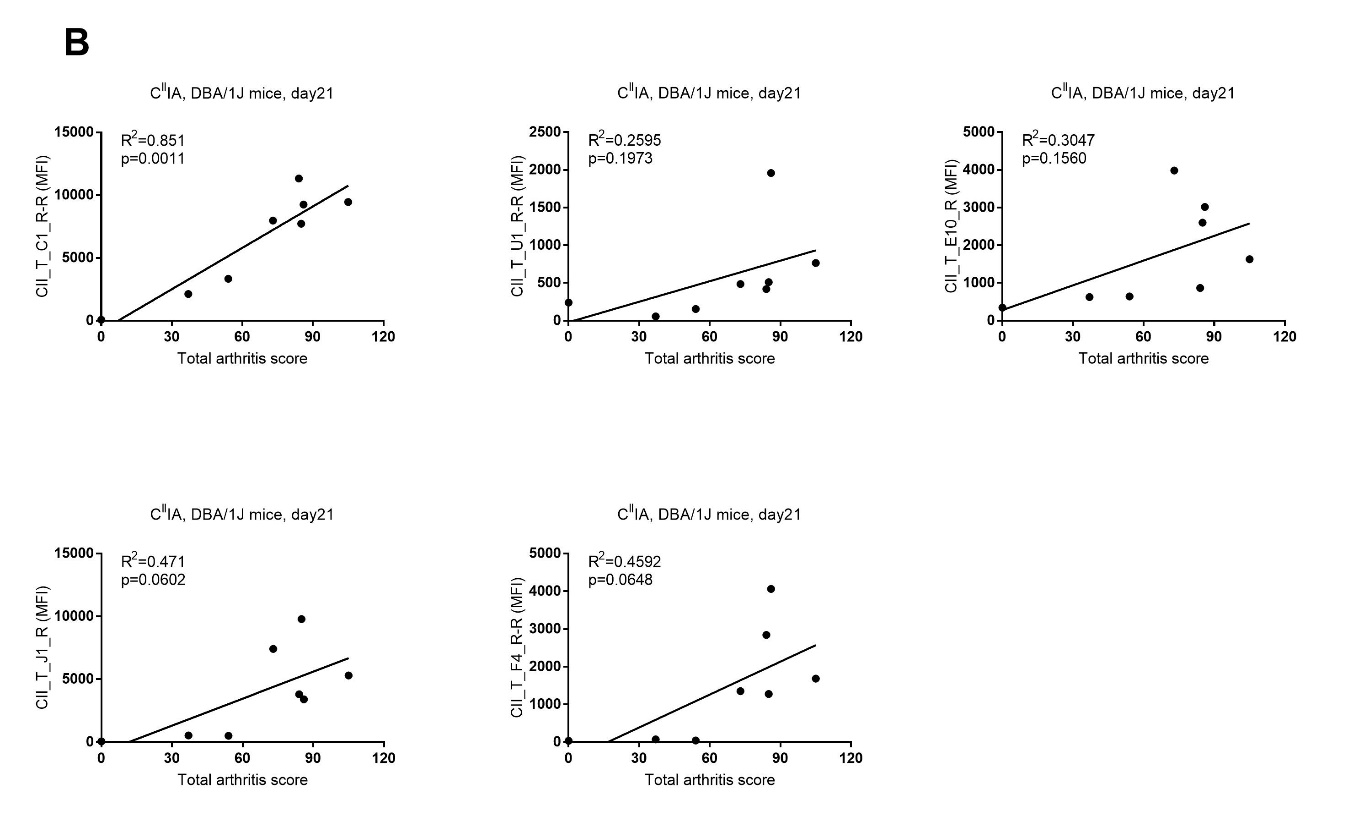
**

**Supplementary Figure 1: Serum antibody response to major CII epitopes and the correlation between epitope response and arthritis severity in C^II^IA mice. (A)** Sera from C^II^IA DBA/1J mice were collected on day 21 and day 70, antibody response to major CII epitopes were measured by bead-based multiplex immunoassays (U1, C1, E10, J1, F4 epitopes). Naïve mice sera were served as negative controls. **(B)** The correlation between serum antibody response to major CII epitopes and total arthritis score in C^II^IA mice (U1, C1, E10, J1, F4 epitopes). Statistics were determined by the Mann-Whitney U test and Pearson correlation test for group comparison and correlation analysis, respectively.


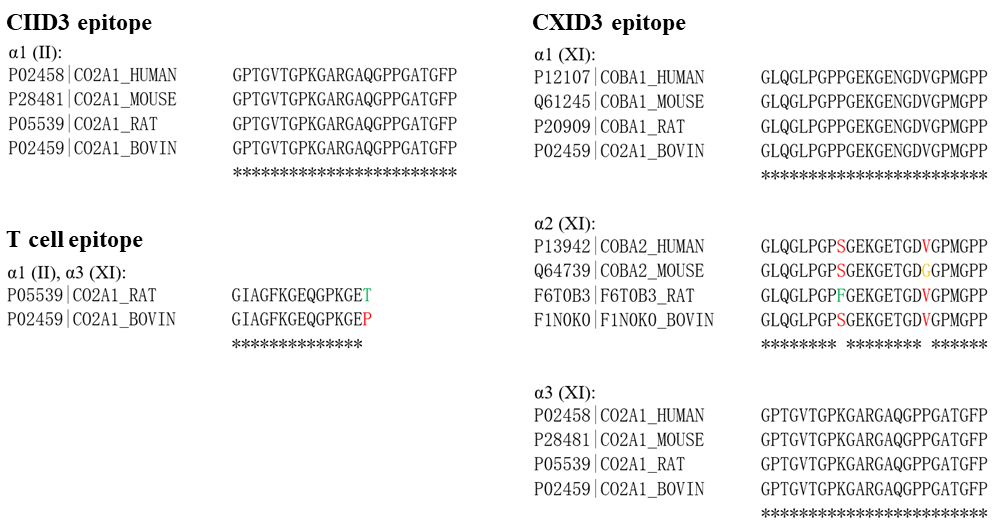


**Supplementary Figure 2:** Sequence alignments for CIID3, CXID3 and T cell epitope among different species. CLUSTAL O (1. 2. 4) multiple sequence alignment was used.
